# Unveiling the full spectrum of maitake mushrooms: A comprehensive review of their medicinal, therapeutic, nutraceutical, and cosmetic potential

**DOI:** 10.1016/j.heliyon.2024.e30254

**Published:** 2024-04-26

**Authors:** Emma Camilleri, Renald Blundell, Bikash Baral, Tomasz M. Karpiński, Edlira Aruci, Omar M. Atrooz

**Affiliations:** a Department of Physiology and Biochemistry, Faculty of Medicine and Surgery, University of Malta, Imsida, MSD2080, Malta; b Centre for Molecular Medicine and Biobanking, University of Malta, MSD2080, Imsida, Malta; c Institute of Biological Resources (IBR), Kathmandu, Nepal; d Chair and Department of Medical Microbiology, Poznań University of Medical Sciences, Rokietnicka 10, 60-806, Poznań, Poland; e Western Balkans University, Autostrada Tirane-Durres km 7, Albania; f Department of Biological Sciences, Mutah University, P.O.Box(7), Mutah, Jordan; g University of Helsinki, Helsinki, Finland

**Keywords:** Maitake mushrooms, *Grifola frondose*, Anti-Cancer, Anti-microbial, Anti-diabetic, Immunomodulation

## Abstract

This literature review provides an up-to-date exploration of the multifaceted attributes of maitake mushrooms (*Grifola frondosa*), elucidating their bioactive phytochemicals and diverse health advantages, including their substantial role in supporting human health and potential incorporation into the medicinal industry. Carbohydrates and protein are the major constituents contributing to the dry weight of *G. frondosa*, taking up around 70–80 % and 13–21 %, respectively, with emerging research linking these constituents to various health benefits. By synthesising current research findings, this review emphasises the substantial role of maitake mushrooms in supporting human health and underscores their potential incorporation into the medicinal industry. To further advance our understanding, future research should delve into the mechanisms underlying their health-promoting effects, with a focus on conducting quantitative studies to elucidate physiological pathways and potential drug interactions. Additionally, exploring their integration into functional foods or nutraceuticals through quantitative assessments of bioavailability and efficacy will be crucial for maximising their therapeutic benefits. This review aims to provide comprehensive insights, catalysing further research and innovation in utilising maitake mushrooms for improved well-being and industry advancement.

## 1 Introduction

The maitake mushroom (*Grifola frondosa*), renowned for its substantial size and belonging to the Polyporaceae family, traces its origins to northern Japan [1]. With a distinctly earthy flavour, captivating aroma, and robust, meat-like texture, it has long been cherished in traditional Asian medicine and cuisine [2]. Notably versatile in culinary applications, maitake mushrooms are prized ingredients in sautés, risottos, and as meat substitutes in Western dishes. Both the fruiting bodies and fungal mycelium of this edible polypore fungus, characterized by overlapping caps and a smoky brown hue [3], boast a rich history of health-promoting benefits, firmly rooted in East Asian traditional medicine. Thus, beyond its culinary versatility, maitake mushrooms have emerged as a beacon of novelty and innovation, inspiring chefs and researchers alike to explore their untapped potential.

While historically treasured for their adaptogenic properties, recognised for enhancing vitality and bolstering the immune system, maitake mushrooms beckon attention for their novel applications and potential benefits [4]. Referred to as "hen-of-the-woods" in traditional Japanese and Chinese medicine and symbolising joy in their discovery ("Dancing Mushrooms") [5], these mushrooms hold cultural significance beyond their medicinal properties. In Chinese tradition, they are also known as "hui-shu-hua" or "grey tree flower," owing to their distinctive appearance [6].

Recent years have witnessed a resurgence of interest in maitake mushrooms, particularly in the realms of alternative medicine and functional foods [7]. Cultivation techniques, ranging from traditional log-based methods to innovative sawdust substrates, testify to the mushroom's adaptability and accessibility on a global scale. Within their unassuming caps lies a treasure trove of bioactive compounds—polysaccharides, glucans, triterpenes, and phenolic compounds—each with their own unique promise. These compounds exhibit not only immunomodulatory and anti-inflammatory properties but also novel anticancer and antioxidant potentials amongst others [[8], [9], [10]]. Therefore, throughout this review, the medicinal benefits and future prospects of *G.frondosa* are highlighted.

## 2 Methodology

### 2.1 Literature search strategy

A comprehensive literature search was conducted to identify relevant studies for the review. The search was carried out in electronic databases including PubMed, Scopus, and Google Scholar. Keywords used included "maitake mushrooms," "*Grifola frondosa,*” "phytochemicals in maitake mushrooms," "health benefits of maitake mushrooms," "anti-cancer and immunomodulating mechanisms of maitake mushrooms," "anti-microbial properties of maitake mushrooms," "anti-diabetic effects of maitake mushrooms," "maitake mushrooms in cosmetics," and related terms.

### 2.2 Inclusion and exclusion criteria

This review included studies centred on investigating the phytochemical composition of maitake mushrooms. It encompassed research elucidating the diverse health benefits of maitake mushrooms, encompassing their anti-cancer, anti-microbial, anti-diabetic, and immunomodulatory effects as well as their potential for skin care applications. Studies selected for inclusion were sourced from reputable journals and were included if the data was sufficient and relevant.

Studies that primarily focused on mushroom species other than maitake were excluded from this review. Non-English articles lacking comprehensive translations were not included. Studies with insufficient relevant data were excluded.

## 3 The geographical distribution of maitake mushroom

The maitake mushroom, widely distributed throughout the Northern Hemisphere [11], thrives primarily in temperate woodlands across Asia, Europe, and North America [4]. Revered in Japan for centuries for its culinary and medicinal qualities, maitake mushrooms also grace the forests of France, Italy, and Poland in Europe [12]. However, they are most commonly found in the northeastern and midwestern regions of North America. Typically flourishing at the bases of hardwood trees such as oak, maple, and elm, maitake mushrooms favour damp, shaded environments [2,3].

## 4 The life cycle of maitake mushroom

**Fig. 1:**
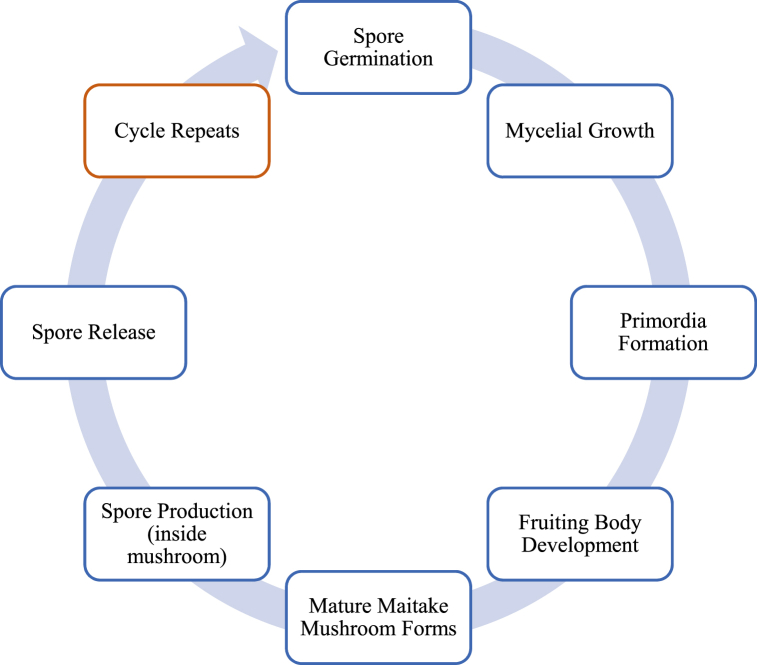
A depicted summary of the maitake mushroom's life cycle.

As shown in Fig. 1, the life cycle of the maitake mushroom epitomises the characteristics of basidiomycetes [13], a group of fungi distinguished by the presence of basidia, specialised structures containing spores. This cycle begins with the sexual reproduction of the maitake mushroom, where haploid spores from two compatible mating strains merge. Germination of these haploid spores initiates the life cycle, leading to the formation of hyphae, branching filaments constituting the fungus's vegetative body. As the hyphae grow and proliferate, they form a network known as mycelium, serving as the structural framework for nutrient absorption [4]. Under favourable conditions, maitake mycelia generate primordia, specialised structures signalling the onset of fruiting. With further differentiation, these primordia develop into mature fruiting bodies, commonly recognised as maitake mushrooms. These mushrooms exhibit distinctive features such as overlapping caps and tightly clustered, downward-hanging, fan-shaped fronds, contributing to their characteristic appearance [14]. During the reproductive phase, basidia located inside the pores or gills on the mushroom's underside undergo meiosis, producing haploid basidiospores [15,16]. The primary mode of dispersal for the species involves the release of these basidiospores into the surrounding environment [17].

## 5 The chemical components of maitake mushroom

**Fig. 2:**
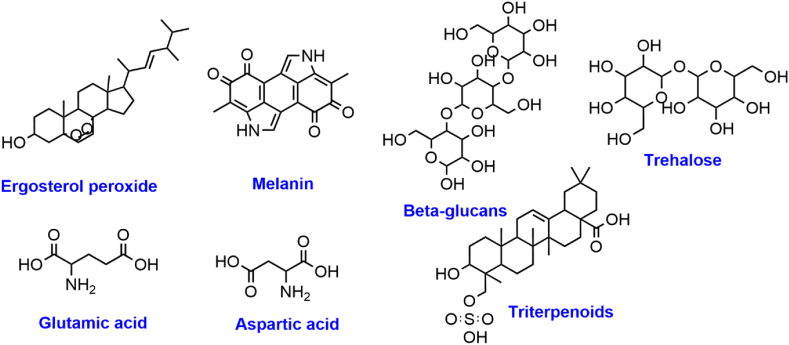
**Some prominent bioactive compounds found in the mycelium and the fruiting bodies of Maitake mushrooms.** It can be appreciated that these bioactive chemicals in Maitake play a pivotal role in contributing to its various medicinal attributes.

The complex chemical makeup of maitake mushrooms adds to their distinct flavour, fragrance, and health advantages. Some components such as trehalose, glutamic acid, aspartic acid, and 5′-nucleotides provide this fungus with its distinctive flavour [18]. As seen in Fig. 2, numerous bioactive substances, including polysaccharides, β-D-glucans, ergosterol, lactulose, dextrin, oligofructose, triterpenes, and different phenolic compounds, are present in maitake mushrooms contributing to their health benefits [2,19]. Additionally having high nutritional value, maitake also has pharmacological properties, such as anticancer activity linked to its β-D -glucan content. These complex carbohydrates possess immunomodulatory activities, which improve the performance of the immune system and have anti-cancer properties [2].

More importantly, maitake synthesises several polysaccharide fractions with anti-inflammatory, anticancer, antiviral, and immunomodulatory properties. Very recently, polysaccharides extracted from this fungus have been found to alter the gut microbiota [20,21], which may also affect immunological homeostasis and result in anticancer effects. Additionally, these polysaccharides may control the makeup of the gut microbiota in the treatment of metabolic diseases and such extracts obtained from this mushroom may act as a probiotic [[21], [22], [23]] Additionally, maitake has proteins and tiny biomolecules with a range of health advantages, such as antioxidant, immune-boosting, anti-diabetic, anti-obesity and other actions [[24], [25], [26]].

Furthermore, when exposed to ultraviolet light, the sterol component ergosterol, which is present in maitake mushrooms, acts as a precursor to producing vitamin D. Triterpenes, a different family of substances found in maitake mushrooms, have anti-inflammatory and anticancer properties, which makes them highly enticing for pharmaceutical study [27,28]. Maitake mushrooms also contain phenolic substances such as flavonoids and phenolic acids, which are known for their antioxidant effects [13]. Numerous health advantages, including cardiovascular protection and anti-ageing effects, have been linked to these substances [29].

Additionally, maitake can degrade lignocellulose by producing different enzymes such as endoglucanases, exogloconases, β-glucosidases, xylanases, lignin peroxidase (LiP), Mn-peroxidase (MnP) and laccases (Lacc) [14]. Thus, Maitake can be used for the biodegradation of different toxic compounds. Maitake has also been shown to have mercury absorption activity [30].

**Fig. 3:**
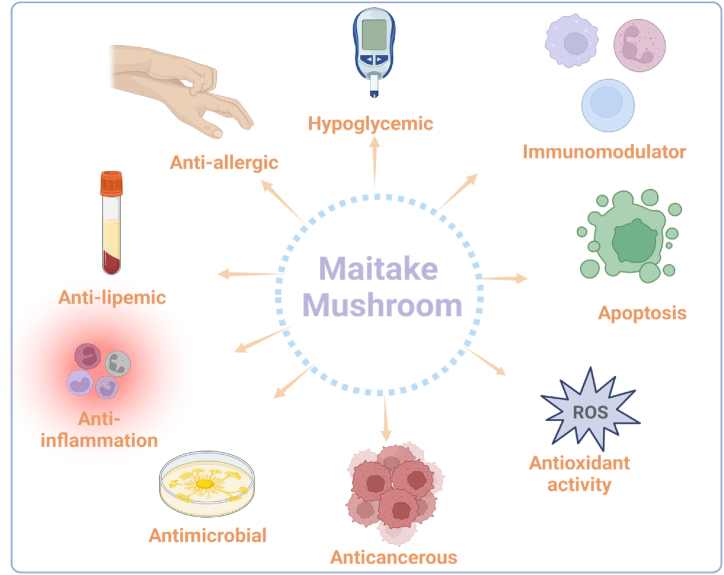
**Utilisation of the extracts of Maitake mushrooms for curing different ailments.** As seen in the image below extracts obtained from Maitake mushrooms can aid in managing several diseases such as cancer, inflammation and allergies.

As depicted in Fig. 3, Maitake mushrooms are a significant source of natural bioactive substances with a range of medicinal uses because of their complex chemical makeup [5]. To completely understand the precise mechanisms and advantages connected with the many chemical components contained in this amazing fungus, further scientific research is required [28]. Recently, the albino mutation in *G. frondosa* was determined to be caused by a single base deletion in the coding region of the tyronisase2 (tyr2) [31]. Because of the lack of undesirable dark brown pigment during processing, these white strains are highly prized for culinary use [31].

## 6 Antimicrobial activity of maitake mushroom

Maitake extract has antibacterial activity (against *S. epidermis* and *P.aeruginosa*), and antifungal activity furanone from maitake (*Pseudallescheria boydii*). Ethanol extracts of *G. frondosa* (rich in organic acids, alkaloids, phenolics, and terpenoids) increased microbiota levels of *Oscillibacter, Butyricimonas, Barnesiella, Turicibacter, Methanosphaera, Asaccharobacter, Globicatella, Bifidobacterium, Allobaculum*, and *Romboutsia* [32]. Despite this, the antimicrobial activity of maitake mushrooms is still poorly understood. This is evidenced by the small number of scientific publications on this subject.

GFAHP, a protein obtained from an extract of *G*. *frondosa* fruiting bodies, has anti-herpes simplex virus (HSV) activity. In plaque reduction assay mean IC_50_ for GFAHP was 4.1 μg/ml and was ten times higher than for acyclovir. The studies found that GFAHP at a concentration of 30 or 60 μg/ml had no effect on the inhibition of HSV-1 attachment but inhibited HSV-1 penetration by 83.7 % and 93.5 %, respectively. Simultaneously, high concentrations of GFAHP (125 and 500 μg/ml) significantly reduced virus production and the severity of HSV-1-induced ocular diseases in animal models [33]. D-fraction obtained from the *G. frondosa* fruiting bodies showed an effect against the hepatitis B virus (HBV). D-fraction inhibited HBV DNA in HepG2 cells with the IC_50_ of 0.59 mg/ml. At the same time, it was found that D-fraction can act synergistically with interferon (IFN) and increase its antiviral activity up to 9 times [34]. A β-glucan (MD-fraction) extracted from *G. frondosa* has a positive effect on HIV patients. 35 patients were administered Maitake tablets containing 250 mg of dried Maitake powder and 5 mg of vitamin C for 12 months. In 20 persons, an increase of CD4^+^ cell amount was reported and in 10 people was reported a decrease in viral load [35]. Ethanolic extract of *G. frondosa* has activity against enterovirus EV71 cultured in Vero cells. The extract inhibited EV71 viral replication, VP1 protein expression, and genomic RNA synthesis. The best activity was observed at 200 μg/ml concentration with an inhibition rate of 88.18 % and IC_50_ of 194.80 μg/ml [36]. Activity against EV71 has also heteropolysaccharide from *G. frondosa* mycelia at the concentration of 250 μg/ml. It inhibits viral replication, VP1 protein expression, and genomic RNA synthesis [37].

In crystal violet assay, *G. frondosa* extract has been shown to inhibit biofilm development by methicillin-resistant *Staphylococcus aureus*. After incubation with *G. frondosa* extract, the average OD value was 0.21 and was significantly lower than the OD in the control group of 0.42 [38]. In Chinese studies, it was found that the water extract of *G. frondosa* has some antibacterial activity. Obtained zones of growth inhibition to *Bacillus subtilis*, *Escherichia coli,* and *S. aureus* were 8.7, 10.2, and 12.4 mm, respectively [39]. Giving into consideration our previous studies, these zones can be assumed to indicate moderate and good (>10 mm) antibacterial activity [40,41]. Also, polysaccharides from *G. frondosa* had an antibacterial effect. In MIC studies first, polysaccharide IPS acted against *S. aureus* (MIC 2.5 mg/ml), *E. coli* (5 mg/ml), *Listeria monocytogenes* (5 mg/ml), and *Bacillus megaterium* (10 mg/ml). The second compound, intracellular zinc polysaccharide, was found to be more effective against the above bacteria, with MICs <0.625 mg/ml, 1.25 mg/ml, 2.5 mg/ml, and 2.5 mg/ml, respectively [42]. It has been shown that D-fraction obtained from the *G. frondosa* increases the survivability of *Listeria monocytogenes*-infected mice. In the control group, all mice died within three days after the bacteria inoculation. After using vancomycin at a dose of 20 mg/kg per day survival rate was higher than 50 %. Administration of the D-fraction at a dose of 10 mg/kg per day improved the survival rate to 60 %. Simultaneously, D-fraction administered at the concentration of 10 mg/kg per day reduced the number of *Listeria* in the peritoneal cavity by 33 % compared to the control sample. The use of vancomycin reduced the number of bacteria by 63 %, and the combination of vancomycin and D-fraction reduced the number of *Listeria* by as much as 94 % [43].

A furanone, grifolaone A, isolated from *G. frondosa,* shows antifungal activity. Potent inhibition of fungal growth was observed against phytopathogens *Fusarium oxysporum*, *Gibberella zeae,* and *Piricularia oryzae* with MIC values of 2.5, 2.5, and 1.25 μg/mL, respectively. For comparison, MICs of carbendazim against the above strains were higher and amounted to 10, 2.5, and 5 μg/mL, respectively. Grifolaone A also had activity against the human pathogen *Pseudallescheria boydii*, with a MIC value of 0.15 μg/mL. This concentration was lower than to amphotericin B (0.31 μg/mL) [44]. Presented above results are shown in Table 1.

**Table 1:** Antimicrobial activity of Grifola frondosa.

Compound/s	Target microorganisms	Active concentration/s	Reference
GFAHP protein	Herpesvirus 1 (HSV)	mean IC_50_ 4.1 μg/ml; at concentrations 30 and 60 μg/ml inhibited HSV-1 penetration by 83.7 % and 93.5 %, respectively; at concentrations 125 and 500 μg/ml reduced virus production	[33]
D-fraction	hepatitis B virus (HBV)	IC_50_ 0.59 mg/ml; synergistic activity with IFN	[34]
β-glucan (MD-fraction)	Human immunodeficiency virus (HIV)	Among 35 patients, in 20 was an increase of CD4^+^ cell amount and in 10 was a decrease in viral load	[35]
Extract	Enterovirus EV71	Inhibition of viral replication, viral VP1 protein expression and genomic RNA synthesis, at the concentration of 200 μg/ml and IC_50_ 194.80 μg/ml	[36]
Heteropolysaccharide	Enterovirus EV71	Inhibition of viral replication, viral VP1 protein expression and genomic RNA synthesis, at the concentration of 250 μg/ml	[37]
Extract	Methicillin-resistant Staphylococcus aureus (MRSA)	inhibition of the MRSA biofilm development, decrease of OD to 0.21 in comparison to the control group of 0.42	[38]
Water extract	*Bacillus subtilis*, *Escherichia coli* and *S. aureus*	zones of growth inhibition were 8.7, 10.2 and 12.4 mm, respectively	[39]
Polysaccharides	*S. aureus*, *E. coli, Listeria monocytogenes*, *Bacillus megaterium*	MICs for polysaccharide IPS 2.5 mg/ml, 5 mg/ml, 5 mg/ml and 10 mg/ml, respectively; MICs for zinc polysaccharide <0.625 mg/ml, 1.25 mg/ml, 2.5 mg/ml, and 2.5 mg/ml, respectively.	[42]
D-fraction	*Listeria monocytogenes*	D-fraction at a dose 10 mg/kg per day improved the survival rate of *Listeria*-infected mice to 60 %; reduction of the number of *Listeria* in the peritoneal cavity by 33 %	[43]
Grifolaone A	*Fusarium oxysporum, Gibberella zeae, Piricularia oryzae, Pseudallescheria boydii*	MIC values of 2.5, 2.5, 0.15 and 1.25 μg/mL, respectively	[44]

## 7 Immunomodulatory and anti-cancer activities of maitake mushroom

As well established *G*. *frondosa* stands as both an edible delicacy and a cornerstone of traditional medicine, with roots dating back centuries [[45], [46], [47]]. Rich in bioactive compounds such as β-glucans and protein units including D-fraction, X-fractions, grilofan, and SX-fraction, this mushroom exhibits potent anti-proliferative and immunomodulatory effects [48]. Numerous studies highlight its ability to activate key immune cells, notably macrophages, natural killer cells (NK), and cytotoxic T cells, pivotal in immune defence and direct tumour cell destruction [49]. Moreover, its glucans stimulate the production of cytokines such as interleukin-1 and interleukin-2, crucial mediators of immune responses [49].

Alonso et al. (2013) reported that the β-glucan component of maitake's D-fraction can influence the switching on and off of genes expressed in human breast cancer Michigan Cancer Foundation (MCF)-7 cells, leading to apoptosis induction. This process controls the breast cancer phenotype and may contribute to the reversal of malignant characteristics. Additionally, other researchers have suggested that maitake extracts exert a direct apoptotic effect on prostate cells, breast cancer cells [50], and kidney cells [51]. Zhao et al. (2017) also noted that combining D-fraction (0.2 mg/mL) and vitamin C (0.3 mmol/L) resulted in a 70 % reduction in human hepatocarcinoma SMMC-7721 cell viability further solidifying maitake's anti-cancer properties [6].

Furthermore, animal studies have demonstrated the safety of maitake D-fraction proteoglucan treatment, showing no toxic effects while providing health benefits and improvements in various types of cancer. These include colon cancer [52], bladder cancer [53], brain cancer, leukemia, liver cancer, breast cancer, and kidney cancer [54]. Additionally, maitake extractions activate effector cells of both the innate [55] and adaptive [56] immune systems, enhancing the production and release of interleukins and lymphokines.

## 8 Antidiabetic activity of maitake mushroom

**Fig. 4:**
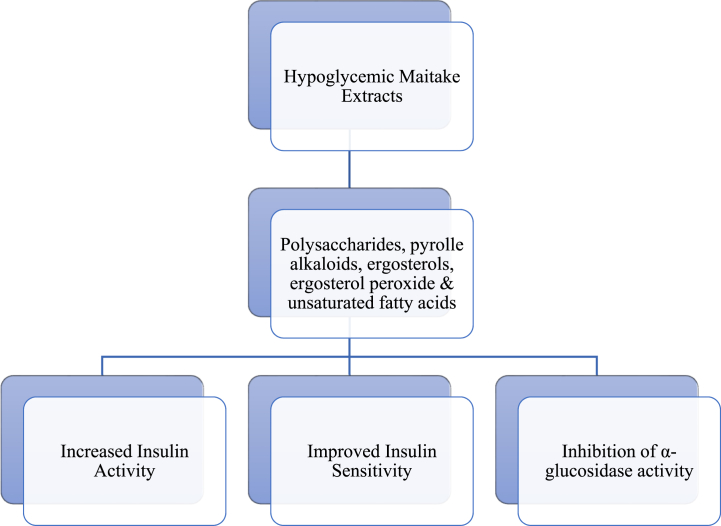
A summary of the hypoglycemic activity of maitake mushrooms.

Animal studies demonstrated that maitake extracts have a hypoglycemic activity. As summarised in Fig. 4, the maitake extract content of polysaccharides, pyrrole alkaloids, ergosterols, ergosterol peroxide, and unsaturated fatty acids play significant roles in the hypoglycemic effect through the insulin signal pathway [57,58]. Previous studies reported that the hypoglycemic effect may be due to the facilitated glucose uptake, leading to the stimulation of insulin receptors (IR, IRS-1), and eventually resulting in increased insulin secretion [58]. Xiao et al. (2012) reported that the polysaccharides of Maitake extract improve insulin sensitivity and decrease fasting serum glucose by increasing protein levels of insulin receptors and decreasing protein levels of insulin receptor substrate 1 [57].

Other studies demonstrated that the antidiabetic activity may be due to the inhibition of α-glucosidase activity [59,60], whereas others suggested that this effect is due to the presence of ergosterols and pyrrole alkaloids in the extracts [60,61]. However, one study suggested that the inhibition of the α-glucosidase activity was attributed to the unsaturated fatty acids the oleic acid and linoleic acid [62].

Additionally, Kubo and Nanba (1997) observed that *G.frondosa*'s fruiting body feed significantly reduced serum triglyceride, cholesterol, and phospholipid levels in rats by 30–80 %, while also lowering liver weight by 60–70 % and increasing faecal cholesterol excretion by 1.8 times. Similarly, Fukushima et al. (2001) reported similar outcomes with *G. frondosa*-fed rats showing lowered serum total cholesterol and VLDL levels, along with increased faecal cholesterol excretion. This further highlights maitake's potential in improving overall health in diabetics since such patients often present with deranged lipid profiles too in conjunction with elevated blood glucose since insulin plays a role in regulating various steps of lipid metabolism.

## 9 Maitake mushrooms’ potential use in skin products

β-Glucans (short β-1,6-branched β-1,3-glucans), found abundantly in fungi, bacteria, yeast, and cereal cell walls, are notable for their diverse health benefits, including anticancer, antioxidative, and anti-inflammatory effects [63,64]. They promote the production of growth factors, collagen biosynthesis, and possess gel-forming properties [65,66]. In skin care, β-glucans are utilised for skin hydration, wound healing, antiaging, and addressing skin burns [67,68]. Studies suggest that β-(1,3)-glucans can mitigate skin cells' oxidative stress and inflammation caused by environmental factors [69]. Maitake extract, rich in β-1,3-glucans, demonstrates antitumorigenesis and anti-carcinogenesis activity [70], while *G. frondosa* mycelium extracts enhance collagen biosynthesis [68]. Polysaccharide-rich extracts also exhibit hypoglycemic and hypolipidemic activities, further highlighting the therapeutic potential of β-glucans [71]. Additionally, Huang et al., 2014 suggest that maitake polysaccharides combined with chitosan can be a promising material for wound healing. Glucans are also used as thickening and stabilising agents in different industries.

Nagao et al., 2009 showed that ethanol extracts of *G*. *aempfer* had the effect of increasing the formation of lipids inside the cells, leading to a higher production of triacylglycerides, and it also triggered the activation of diacylglycerol acyltransferase. Maitake ethanolic extract can aem be used for different skin problems associated with dry skin or xeroderma such as atopic dermatitis [72]. Other studies suggest that *G*. *frondosa* polysaccharides have inhibitory effects on melanogenesis and can be used as skin-whitening agents.

**Fig. 5:**
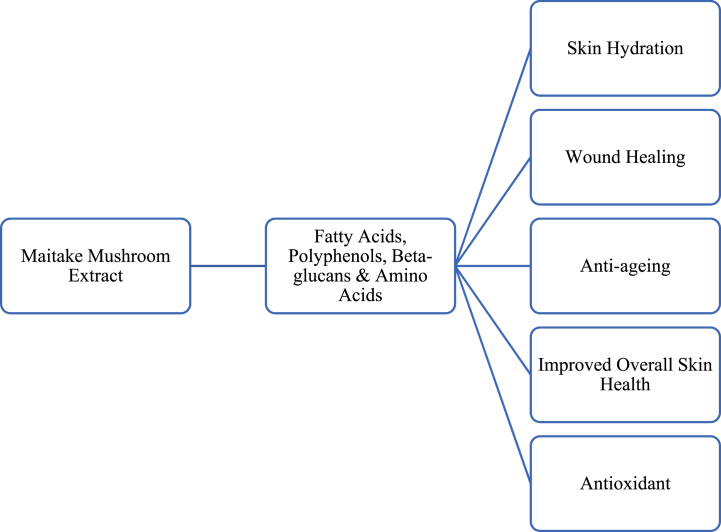
A visual overview of the potential of maitake mushrooms' bioactive compounds in skin products.

Moreover, the *G*. *aempfer* extracts have been demonstrated to represent an important source of bioactive compounds with antioxidant activities. In fact, Zhang et al. (2002), demonstrated that fatty acids in *G. aempfer* inhibited cyclooxygenase (COX)-1 and COX-2 enzyme activities by 98 % and 99 %, respectively, and showed 79 % inhibition of liposome peroxidation, emphasising its significant antioxidant and anti-inflammatory properties [6]. Additionally, polyphenols with known antioxidant activities found in maitake are tannic acids, gallic acids, flavonoids (naringenin, hesperidin, pseudobaptigenin, cyaniding 3-*O*-xylosylrutinoside) [73] chlorogenic acid, and kaempferol [74]. Sharpe et al., 2021 tested the antioxidant activity of six mushroom species (maitake, Chaga, shiitake, reishi, turkey tail, and lion's mane) and showed that the higher antioxidant activity was found in chaga and maitake. Ziewlewska et al., 2023 showed that maitake extracts have higher polyphenol, flavonoid content, and antioxidant activity compared with reishi and lion's mane extracts. Ji et al., 2019 showed that maitake alcohol-soluble polysaccharides have excellent antioxidant activity. Maitake contains also a high quantity of ergothioneine sulfur-containing amino acid with antioxidant activities. In saying this, maitake's antioxidant properties make it a viable and potential component that can be incorporated into skin care products as seen in Fig. 5.

Furthermore, terpenoids and sterols are found in maitake extracts. Fatty acids produced by maitake are palmitic, oleic, linoleic acids, ergosterol, and ergosterol peroxide. Amino acids found in maitake are l-Leucine, l-alanine, l- Arginine, l- Aspartic acid, l-Glutamic acid, GABA, glycine, l-histidine, l-Isoleucine, l-leucine, l-lysine, l-methionine, l-Phenylalanine, l-serine, l-threonine, l-tryptophan, l-tyrosine andL-valine [6]. The composition of fatty acids and amino acids in maitake mushrooms holds promise for the development of cosmetic products aimed at maintaining and repairing the skin's hydrolipidic film.

## 10 Conclusion

Conclusively, this comprehensive review has demonstrated the multifaceted attributes and promising potential of maitake mushrooms across medicinal, therapeutic, nutraceutical, and cosmetic realms. The evidence presented underscores their effectiveness in combatting various health conditions, spanning from cancer and diabetes to immune disorders and skin ailments. Notably, key bioactive components, particularly polysaccharides, play pivotal roles in eliciting diverse health benefits, including antitumor, immunomodulatory, antimicrobial, anti-inflammatory, antidiabetic, lipid metabolism regulation and antioxidative effects.

Looking forward, future research efforts must prioritise the complete understanding of the physiological mechanisms responsible for maitake mushrooms' therapeutic properties. Moreover, conducting robust clinical trials to assess potential side effects and drug interactions in human subjects is imperative. Quantitative evaluations of these parameters will not only deepen our understanding of maitake product safety but also guide healthcare providers and consumers in making informed decisions. Additionally, gauging consumer and medical practitioner acceptance of maitake-based products will be pivotal for their seamless integration into mainstream healthcare practices.

Furthermore, in light of growing concerns about health threats such as food contamination, environmental pollution, and emerging infectious diseases like the COVID-19 virus, leveraging *G. frondosa*'s immunomodulatory and health-promoting functions holds significant promise for safeguarding human health. Thus, in summary, by persistently exploring maitake mushrooms' medicinal and therapeutic potential through quantitative research methodologies and rigorous clinical trials, one can foster widespread acceptance and utilisation, thereby enhancing human health and well-being.

## Funding

This research did not receive any specific grant from funding agencies on the public, commercial, or not-for-profit sectors.

## Ethics declaration

Review and/or approval by an ethics committee as well as informed consent was not required for this study because this literature review only used existing data from published studies and did not involve any direct experimentation/studies on living beings.

## Data availability statement

No data was used for the research described in the article. No data associated in this article has been deposited into a publicly available repository.

## CRediT authorship contribution statement

**Emma Camilleri:** Writing – review & editing, Supervision, Project administration, Conceptualization. **Renald Blundell:** Supervision, Conceptualization. **Bikash Baral:** Writing – original draft. **Tomasz M. Karpiński:** Writing – original draft. **Edlira Aruci:** Writing – original draft. **Omar M. Atrooz:** Writing – original draft.

## Declaration of competing interest

The authors declare that they have no known competing financial interests or personal relationships that could have appeared to influence the work reported in this paper.
